# Molecular analysis of *reticulocyte binding protein-2* gene in *Plasmodium vivax* isolates from India

**DOI:** 10.1186/1471-2180-12-243

**Published:** 2012-10-24

**Authors:** Surendra K Prajapati, Pragati Kumari, Om P Singh

**Affiliations:** 1 Molecular Biology Division, National Institute of Malaria Research (NIMR), Sector 8, Dwarka, Delhi, India

## Abstract

**Background:**

*Plasmodium vivax* reticulocyte binding protein-2 (PvRBP-2) is a promising candidate for development of vaccine against parasite. DNA sequence polymorphism in *pvrbp-2* which may hamper the vaccine development program has been identified in laboratory strains. Therefore, unraveling genetic polymorphism in *pvrbp-2* from field isolates is a prerequisite for success in vaccine development. This study was designed with a primary aim to uncover genetic polymorphism in *pvrbp-2* among *P. vivax* field isolates.

**Results:**

Using virtual restriction mapping of *pvrbp-2* sequences, two restriction enzymes (*Alu*I and *Apo*I) were selected for the development of *pvrbp-2* as a PCR-RFLP marker. Restriction fragment length polymorphism (RFLP) analysis revealed a high degree of genetic polymorphism in the *pvrbp-2* gene among field isolates of *P. vivax*. *Apo*I-RFLP was found to be more efficient in identifying the extent of genetic polymorphism in *pvrbp-2* compared to *Alu*I-RFLP. Combined genotyping/haplotyping of RFLP pattern revealed a total of 36 distinct RFLP patterns among 83 *P. vivax* isolates analyzed. DNA sequence analysis also supports high degree of genetic polymorphism among field isolates of *P. vivax*. *Pvrbp-2* PCR-RFLP method is able to distinguish multiple infection up to 16.86% and it revealed a low level of shared genetic pool between more than two populations.

**Conclusion:**

The study suggests that *pvrbp-2* is highly polymorphic genetic marker which can be used for population genetic analyses. RFLP analysis suggests presence of nearly similar proportion of *Sal-1* and *Belem* alleles in Indian *P. vivax* populations. The larger extent of genetic polymorphism identified from limited samples advocates to screen genetic polymorphism in *pvrbp-2* from malaria endemic geographical regions and countries for designing *pvrbp-2* based anti-malarial control measures.

## Background

*Plasmodium vivax* is the most widely distributed human malaria parasite outside sub Sahara regions of Africa. Although mild with its prolonged and recurrent infection resulting in huge morbidity, the species can also be severe and fatal [1-6]. Annual burden is estimated to be about 70–80 million cases globally [7], however in India, *P. vivax* is responsible for about one million malaria cases annually, contributing 50–55% of total malaria cases.

Using molecular techniques, genetic diversity studies of malaria parasites accelerated substantially and provided a landmark in understanding parasite genetic diversity, evolution of pathogenicity and drug resistance, and transmission success. Identifying highly polymorphic marker is essential for studying genetic diversity, population structure, multiplicity of infection, and relapse and recrudescence infection etc. Till date, two types of molecular markers are in frequent use to unraveled genetic diversity from field isolates of *P. vivax*, these are tandem repeats markers [8,9] and antigen encoding genes [10-12].

Invasion of erythrocytes by malaria parasite is a complex and multi-step process. Merozoites of *P. vivax* primarily invade the reticulocytes [13] whereas *P. falciparum* can invade both mature RBC as well as reticulocytes [14,15]. The specificity in binding with reticulocytes is mediated by a set of proteins which are encoded by a gene family called *reticulocyte binding protein* where members of this family are found in malaria parasites of human, simian and rodent [16-19]. The major function of reticulocyte binding protein is seen during the initial steps of erythrocyte selection and invasion [17]. Evidence suggests that the PvRBPs form a complex at the apical pole of the merozoite and confer the reticulocyte-specificity of *P. vivax* blood-stage infections, suggesting the essential role of RBP-II in selection and identification of reticulocyte for invasion [17]. Two *pvrbp-2* genes have been characterized from *P. vivax* and are shown to be a promising vaccine candidate [20]; however, up to 12 putative *pvrbp* genes have been identified in *P. vivax* genome so far [21].

*Pvrbp-2* is a promising vaccine target for the development of effective anti-malarial control measures [20]. However, genetic polymorphism at *pvrbp-2* may hamper the efficacy of vaccine [22]. Therefore, investigation of genetic polymorphism at *pvrbp-2* from geographical field isolates is an essential step. This study was designed to investigate the genetic polymorphism in *pvrbp-2* using PCR-RFLP method in *P. vivax* field isolates from Indian subcontinent.

## Methods

### Ethics statement

This study was approved by the Ethics Committee of the National Institute of Malaria Research and all blood spots were collected with written consent of the patients and/or their legal guardians.

### Parasite collection and DNA extraction

**Figure 1:**
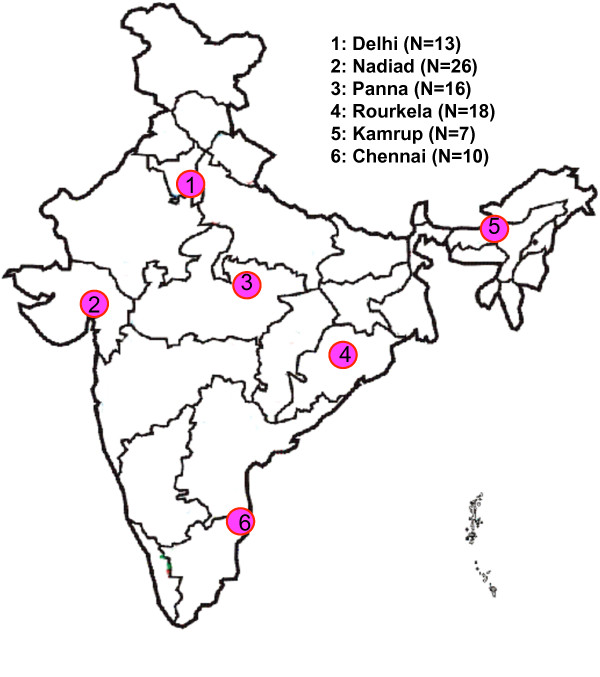
Map of India showing study sites. N indicates number of sample from individual geographical region.

Ninety *P. vivax* field isolates collected between 2003–2006 from six geographical regions of the Indian subcontinent were analyzed (Figure 1). Finger prick blood from the symptomatic patients in active case detection surveys as well as from patient attending the clinics, was spotted on autoclaved Whatman filter paper strips (Number 3). The six geographical regions are Delhi (N=13), Nadiad of Gujarat (N=26), Panna of Madhya Pradesh (N=18), Rourkela of Odisa (N=16), Chennai of Tamil Nadu (N=10), and Kamrup of Assam (N=7). Details of individual study sites such as location, parasite and vector species prevalence, and disease transmission pattern are reported elsewhere [23] as well as given in Additional file 1. Genomic DNA was isolated from microscopically diagnosed vivax-positive blood spotted on Whatman filter paper (3 mm) strips using QIAamp mini DNA kit (Qiagen, Germany). Three punches (5 mm diameter) of dried blood spots were used for DNA isolation, as per the manufacturer’s instructions. DNA was eluted in 120 μl triple distilled autoclaved water and stored at −20°C for future use.

### Primer designing and PCR amplification

**Figure 2:**
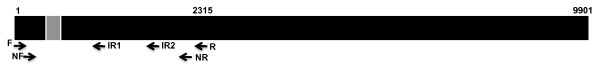
**Diagrammatic representation of primers location on *pvrbp-2 *gene.** Gray and black boxes indicate intron and exon respectively, and arrows indicate location of primers. F and R: forward and reverse primers of primary PCR respectively, NF and NR: forward and reverse primers of nested PCR respectively. IR1 and IR2 are internal sequencing primers.

Nested PCR primers for *pvrbp-2* gene were designed manually using *pvrbp-2* sequence available in GenBank (AY501887). These primers are RBP2-F (5’-gatgatcaatttttatgcctgac-3’), RBP2-R (5’-cagaatccgcaataatagag-3’), RBP2-NF (5’-ttcccgcacacacaaggtag-3’), RBP2-NR (5’-gcgtagtgtttagctgccac-3’), RBP2-IR1 (5’-tggaaccgtatgcgattc-3’) and RBP2-IR2 (5’-ttttgcagataagatagc-3’). Internal primers used for sequencing this fragment are IR1 and IR2 and the schematic diagram of gene showing location of primers is given in Figure 2. Optimized PCR conditions for primary PCR for amplification of *pvrbp-2* were:-initial denaturation 95°C/5 minute, denaturation 95°C/30 S annealing 50°C/30 S and extension at 68°C/2 minute for 35 cycles, and a final extension of 68°C/5 minute. The cycling conditions of nested PCR were similar to primary PCR except annealing temperature, which was 55°C. All PCR amplifications were carried out in a 20 μl reactions volume (Qiagen’s Master Mix) with 10 pM of each primer and.1-2 μl (~ 3–5 ng) of genomic DNA in primary PCR and 0.5-1 μl of primary PCR product in nested PCR.

### Restriction Fragment Length Polymorphism (RFLP)

To determine the level of *pvrbp-2* polymorphism, RFLP analysis was carried out using two restriction enzymes *Apo*I and *Alu*I (NEB Inc, USA). These enzymes were selected on the basis of maximum probability of enzymes cutting sites in the polymorphic region of *pvrbp-2* and the feasibility to resolve digested PCR fragment on agarose gel. Virtual restriction mapping of *pvrbp-2* was done using SeqBuilder module of DNA Lasergene 7.1 software for identification of suitable restriction enzymes for RFLP study. Four microliters of PCR product was digested with individual restriction enzyme. *Alu*I digestion was incubated at 37°C for 4 hours whereas *Apo*I was incubated at 50°C for overnight. In both digestions, heat inactivation for enzymes was given at 80°C/20 minutes. The restriction products were visualized on a 2.5 % agarose gel containing ethidium bromide. A consistent current at 0.75 m for 2.5 hrs were used for all agarose gel electrophoresis experiments to achieve consistency in RFLP fragment sizes.

### RFLP Genotyping and multiple infection typing

Digested DNA fragments were assessed using Genetool software and all fragments were considered for genotyping of RFLP data. In RFLP analysis, the restriction pattern of each enzyme was typed where each different/unique RFLP pattern was assigned 1…n as an allele. Finally, RFLP patterns of *Apo*I and *Alu*I from each sample were combined to make a “haplotype or genotype”. This “haplotyping/ genotyping” method provides a high-resolution power for differentiating parasites compared with RFLP pattern of individual enzyme.

Multiple infection could only be detected by RFLP analysis since all samples show only a single PCR fragment. A sample was considered as multi-clone infection if the sum of the digested fragments (either *Apo*I or *Alu*I or both) size is greater than the size of the PCR fragment.

### Cloning, DNA sequencing, and sequence analysis

DNA sequencing of limited samples was done in order to validate RFLP pattern as well as to differentiate *Sal-1* and *Belem* alleles of *pvrbp-2*. PCR products from 13 samples (Nadiad; 7, Delhi; 1, Kamrup; 2, and Panna; 3) were purified using gel extraction kit (MDI, India) and cloned in pTZ257R/T vector (Fermentas, USA). Six of 13 samples were single clone in nature on the basis of *pvrbp-2* RFLP analysis. Plasmid was purified using plasmid extraction kits (MDI, India) and purified plasmids were sequenced commercially (Macrogen Inc, Seoul, Korea) [24]. For DNA sequencing, each plasmid was sequenced with forward, reverse and internal primers. DNA Lasergene software 7.1 (DNA Star Inc., USA) was used for editing raw DNA sequences (EditSeq module), with SeqMan module used for contig formation and ClustalW module for sequences alignment. DNA sequences of *pvrbp-2* obtained from field isolates of *P. vivax* were deposited in GenBank (JN872360-JN872372).

## Results

### Identification of genetic polymorphism using PCR-RFLP method

**Figure 3:**
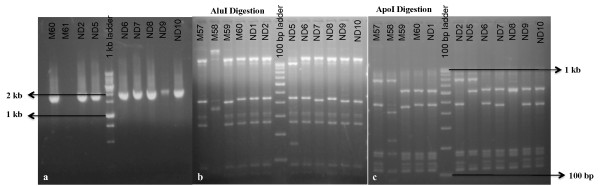
**Gel pictures of PCR and RFLP of *pvrbp-2 *gene, a) PCR amplification, b) *Alu*I digestion, c) *Apo*I digestion.** Name above the each well represents sample identity.

A total of 90 *P. vivax* samples were analyzed where in all samples gave single clear amplification of ~2.0 kb fragment size and none of the PCR fragments showed size variation (Figure 3a). Amplified PCR fragment covers both coding and non-coding regions. The coding regions are marked by a 449–503 bp and 705–1946 bp in the amplified PCR product (Figure 2). Virtual restriction mapping of *pvrbp-2* sequence suggests the use of *Apo*I and *Alu*I restriction enzymes for RFLP analysis. Initially, five samples were digested with the above two enzymes to make sure that these enzymes can identify genetic polymorphism from field isolates. Interestingly, genetic polymorphism in *Alu*I and *Apo*I digestion was observed in selected five samples. Further, PCR products obtained from 83 *P. vivax* isolates were digested with *Alu*I and *Apo*I enzymes separately. RFLP pattern of *pvrbp-2* gene with *Alu*I and *Apo*I has been shown in figure 3b & c respectively.

**Figure 4:**
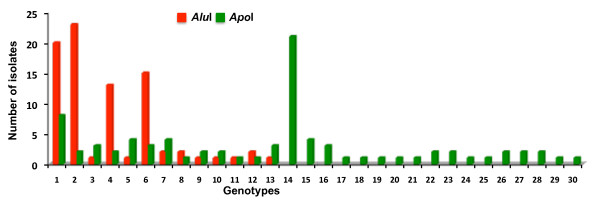
**Distribution of *Alu*I and *Apo*I RFLP genotypes among Indian field isolates of *Plasmodium vivax*.**

A substantial number of RFLP pattern for both enzymes were observed with respect to the Sal-1 strain based *pvrbp-2* gene sequence. In total, 13 distinct *Alu*I and 30 distinct *Apo*I RFLP patterns were observed among 83 samples. RFLP analysis revealed mainly two distinct digestion patterns in field isolates by both enzymes. This suggests that each enzyme has two major types of digestion pattern. RFLP pattern of six samples was confirmed by DNA sequencing. Among six samples, DNA sequences of five samples were in agreement with RFLP data, however in a single sample (Pv-7) RFLP pattern of only *Apo*I enzyme was not matched. This may be due to the sequencing of only one clone from each cloning experiment. The numbers of RFLP pattern of individual enzymes from all samples are listed in Table 1. The frequencies of *Alu*I and *Apo*I genotypes varied in field isolates (Figure 4). Further, combination of *Alu*I and *Apo*I RFLP patterns revealed a total of 36 distinct haplotypes/genotypes suggesting a high degree of genetic diversity in *pvrbp-2* sequences in the field isolates of *P. vivax*.

**Table 1:** **RFLP data and genotyping of *Plasmodium vivax *using *pvrbp-2 *gene**

**Sample**	***Alu*I RFLP Pattern (bp)**					***Alu*I Genotype**	**Combined Genotype/number of isolates**	***Apo*I Genotype**	***Apo*I RFLP Pattern (bp)**							
Pv-2	1200	410	280	90		1	1/8	1	650	450	180	170	160	140	120	80
Pv-7	1200	430	280	90		2	2/2	2	650	180	170	160	130	120		
Pv-14	1500	1200	410	380	280	3	3/1	3	800	650	450	410	250	180	120	
ND-52	1500	380	80			4	4/2	4	800	410	250	180	120	90		
ND-51	1200	430	280	90		2	5/5	5	650	480	180	170	160	140	130	120
ND-54	1200	410	280	180	90	5	6/1	6	650	450	180	170	160	140	130	120
M-53	1500	380				6	7/3	7	800	410	250	180	120			
M-54	1200	430	380	90		7	8/1	7	800	410	250	180	120			
M-55	1200	430	280	90		2	9/1	8	850	800	750	180	170	120	80	
ND-57	1000	430	280	180	90	8	10/2	9	800	650	180	170	120			
RR-1	1200	430	280	90		2	11/2	10	800	480	180	170	140	120	80	
RR-18	1200	430	380	80		7	12/1	11	650	450	250	180	160	120	90	
M-51	1200	430	280	90		2	13/1	12	650	180	170	160	130	120	80	
M-57	1200	410	280	90		1	14/2	13	800	480	180	170	140	120		
M-58	1500	380				6	15/10	14	800	410	250	180	120	80		
M-59	1200	410	280	90		1	16/4	15	650	450	180	170	160	140	120	
ND-1	1200	430	280	90		2	17/3	16	650	480	180	170	160	140	120	
ND-2	1200	430	280	90		2	18/1	13	800	480	180	170	140	120		
ND-8	1200	430	280	90		2	19/1	17	650	180	170	160	120			
ND-12	1200	430	280	90		2	20/1	18	800	650	180	170	130	120		
ND-14	1200	430	410	280	90	9	21/1	19	800	650	450	180	170	140	130	120
ND-29	1500	380	80			4	22/10	14	800	410	250	180	120	80		
P-5	1500	380	80			4	23/1	21	650	410	250	180	160	120	80	
R-12	1200	410	280	90		1	24/2	22	650	600	180	170	160	120		
R-18	1200	430	280	90		2	25/2	23	800	480	180	170	140	130	120	
A-385	1500	1200	430	380	280	10	26/1	24	800	650	410	250	180	170	120	
R-37	1200	430	280	90		2	27/1	25	800	650	480	180	170	120		
D1	1200	410	280	90		1	28/2	6	650	450	180	170	160	140	130	120
D5	1200	430	280	90		2	29/2	26	650	600	180	170	160	130	120	
S2	1500	380				6	30/2	27	800	650	250	180	120			
S4	1200	430	280	90		2	31/1	28	650	450	180	170	160	140	130	120
C1	1200	410	280	90		1	32/1	29	600	180	170	160	130	120		
C2	1200	410	280	90		1	33/1	28	600	450	180	170	160	140	130	120
C5	1200	430	380			11	34/1	30	800	450	250	180	120	80		
C6	1500	1200	430	410	280	12	35/2	3	800	650	450	410	250	180	120	80
C8	1200	400	380			13	36/1	14	800	410	250	180	120	80		

### Multiple infection and population genetic structure

In brief, if a mono-infection sample (infection of single species) show a single genotype, it is denoted as single-clone infection, but if it shows more than one genotype, it is denoted as a multi-clone/multiple infection. RFLP analysis using *Alu*I, showed seven isolates (8.43%) to have multi-clone infection. In contrast, *Apo*I showed 13 isolates (15.66%) as multi-clone infection. In total, 14 isolates (16.86%) were observed to have multi-clone infection. Four multi-clone samples were having both *Sal-1* and *Belem* alleles, however remaining ten multi-clone isolates were infected with different genotypes of either *Sal-1* or *Belem* alleles.

**Figure 5:**
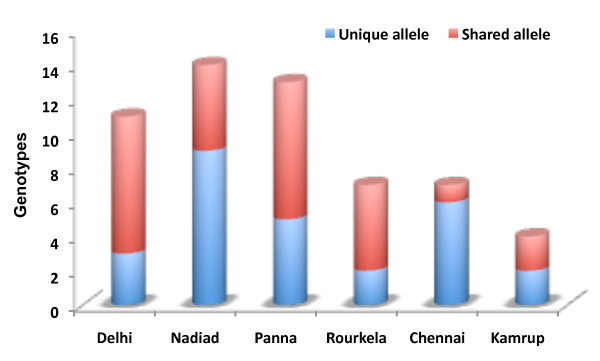
**Number of unique and shared *pvrbp-2 *genotypes among study sites.**

Genetic polymorphism was observed among all geographical regions of the Indian subcontinent. The total number of genotypes observed in Delhi, Nadiad, Panna, Rourkela, Chennai and Kamrup were 11, 14, 12, 7, 7 and 4 respectively. In every geographical region, genotypes were observed to be unique (local polymorphism) and shared in varied proportion (Figure 5). Allelic analysis shows a limited fraction of genotypes were observed to have been shared within 2–3 populations. Only a single genotype (14) was observed in five geographical regions, however, none of the shared genotypes was observed between six geographical regions. This study suggests a diverse pool of *pvrbp-2* repertoire in all geographical regions. This study also uncovered many unique *pvrbp-2* genotypes to exist among geographical regions.

### DNA sequence polymorphism

To understand and support the high degree of genetic diversity observed in PCR-RFLP analysis in *pvrbp-2*, 13 random samples (Nadiad; 7, Delhi; 1, Kamrup; 2, and Panna; 3) were sequenced, of which six isolates were RFLP analyzed. DNA sequence analysis also revealed a high degree of genetic polymorphism such as indels/tandem repeats and single nucleotide polymorphism (SNPs) among field isolates of *P. vivax*. Two indels were found which were restricted to non-coding region. The tandem repeat consisted of six amino acids (PA/TT/VQKK) revealed as 0–3 repeats in field isolates. A total of 178 SNPs were found, out of which 32 were in non-coding region while the remaining were in coding region. The observed higher number of SNPs was mainly due to the dimorphism between *Sal-1* and *Belem* type alleles. Number of non-synonymous substitutions in coding region was higher (n=106) as compared to synonymous substitutions (n=46), which indicates that *pvrbp-2* is under positive selection pressure. None of the SNP (synonymous or non synonymous) was associated with frame shift mutation. Comparison of *pvrbp-2* sequences from Indian field isolates with *pvrbp-2* reference sequence (Sal-1: *P. vivax* strain) suggests a higher degree of DNA sequence polymorphism.

### Distinguishing *Belem* and *Sal-1* alleles with RFLP

The virtual restriction mapping of *pvrbp-2* sequences *Alu*I and *Apo*I enzymes reveals a distinct RFLP pattern of *Belem* and *Sal-1* alleles. Virtual restriction mapping of *pvrbp-2* with *Alu*I revealed a distinct 1500 bp and 380 bp fragments for *Belem* allele. Similarly, virtual restriction mapping with *Apo*I showed a distinct 250 bp fragment for *Belem* allele. The results of virtual restriction mapping of *Belem* and *Sal-1 pvrbp-2* sequences with *Alu*I and *Apo*I enzymes were confirmed with RFLP analysis of field isolates. On the basis of RFLP patterns, all samples were categorized according to the *Sal-1* and *Belem* type. Of the 83 *P. vivax* isolates analyzed, 38.55% (32/83) were *Belem* type, 56.63% (47/83) were *Sal-1* type, and 4.82% (4/83) were mixed of both alleles (Table 2). Furthermore, comparison of RFLP pattern showed *Sal-1* alleles to be more polymorphic (24/36) than *Belem* allele (12/36) in the natural parasite populations. Thus, dimorphism observed in sequence analysis could also be identified by simple PCR-RFLP method.

**Table 2:** **Distribution of *Pvrbp-2 *based *Sal-1 *and *Belem *alleles in field isolates**

**Geographical regions**	**Sample size (N)**	***Sal-I***	***Belem***	**Both**
Delhi	13	8	5	
Nadiad	21	17	4	
Panna	18	7	11	
Rourkela	16	10	4	2
Chennai	10	3	5	2
Kamrup	5	2	3	
Total (n)	83	47	32	4

## Discussion

Malaria eradication program is facing remarkable challenges due to spread of drug resistance and the complex population genetic structure of human malaria parasites. Gaining an insight into the genetic population structure of the parasites would provide valuable information for designing an improved malaria control strategy. The present study investigates genetic polymorphism in *pvrbp-2* among field isolates of *P. vivax* using simple PCR-RFLP.

This is the first population based study of *pvrbp-2* gene which revealed a high degree of polymorphism in field isolates of *P. vivax*. The sequence polymorphism reported in *pvrbp-2* from four strains of *P. vivax* including Sal-1 and Belem [22] is supporting the extent of genetic polymorphism observed in *pvrbp-2* in Indian isolates. The sequences of *pvrbp-2* have shown a distinct dimorphism between *Sal-1* and *Belem* alleles [22]. The dimorphism between Sal-1 and Belem strains of *P. vivax* has been reported earlier on the basis of *pvmsp-1*[25] and the distinction between Sal-1 and Belem strains is entirely based on geographical location and allelic variation. The RFLP analysis of the present study using *Alu*I and *Apo*I enzymes revealed a high degree of genetic polymorphism among field isolates which was further supported by *pvrbp-2* nucleotide sequence polymorphism data. From RFLP analysis, it is clear that *Apo*I is identifying larger extent of genetic polymorphism in field isolates compared to *Alu*I. This suggests that under limited resources, *Apo*I alone can be used to resolved larger extent of existing genetic variation in *pvrbp-2* in the field isolates*.* The genetic polymorphism displayed by various antigen-encoding genes and biochemical marker in Indian field isolates of *P. vivax*[26-32] is also supported by the genetic polymorphism observed in *pvrbp-2*.

*Plasmodium vivax* isolates from Indian subcontinent represents diverse pool of genetic variants such as *Belem* and *Chesson* alleles in *pvgam-1*[23], *Belem* and *Sal-1* alleles in *pvmsp-1*[30], and *VK210* and *VK247* in *pvcsp*[30]. Though, *pvrbp-2* based *Sal-1* and *Belem* alleles have not been identified from natural parasite populations, however present study uncovered both alleles in Indian *P. vivax* populations. As like other above genetic markers, *pvrbp-2* also harbors both *Sal-1* and *Belem* alleles in Indian populations however, their proportion varied between geographical regions.

*Pvrbp-2* is a promising vaccine target for the development of effective anti-malarial control measure [20]. Identifying allelic polymorphism in *pvrbp-2* within and between populations would certainly improve and extend the existing knowledge for development of anti-malaria control measure. The significance of this prospective study would be to uncover maximum number of hidden polymorphism. Several studies in recent past have shown many polymorphic forms in local population [10,12,31,33]. This study revealed genetic polymorphism in *P. vivax* populations which have been rarely shared between more than two populations which suggests that in the natural population, *pvrbp-2* is diverse and this calls for thorough care to be taken while designing any anti-malarial strategy targeting *pvrbp-2.*

## Conclusions

The study suggests that *pvrbp-2* is highly polymorphic genetic marker which can be used for population genetic analyses. RFLP analysis suggests presence of nearly similar proportion of *Sal-1* and *Belem* alleles in Indian *P. vivax* populations. The larger extent of genetic polymorphism identified from limited samples advocates to screen genetic polymorphism in *pvrbp-2* from malaria endemic geographical regions and countries for designing *pvrbp-2* based anti-malarial control measures.

## Competing interests

Authors declare that they don't have competing interests.

## Author’s contribution

SKP: Conceptual designing, experimental design and work, data analysis and manuscript writing, PK: Experimental work and data compilation, OPS: Overall supervision and manuscript writing. All authors read and approved the final manuscript.

## Supplementary Material

Additional file 1 Detail information about study sites. Click here for file
